# Cross-Sectional Study on MRI Restaging After Chemoradiotherapy and Interval to Surgery in Rectal Cancer: Influence on Short- and Long-Term Outcomes

**DOI:** 10.1245/s10434-018-07097-7

**Published:** 2018-12-13

**Authors:** Robin Detering, Wernard A. A. Borstlap, Lisa Broeders, Linda Hermus, Corrie A. M. Marijnen, Regina G. H. Beets-Tan, Willem A. Bemelman, Henderik L. van Westreenen, Pieter J. Tanis, Dutch Snapshot Research Group, A. Aalbers, Y. Acherman, G. D. Algie, B. Alting von Geusau, F. Amelung, S. A. Bartels, S. Basha, A. J. N. M. Bastiaansen, E. Belgers, W. Bleeker, J. Blok, R. J. I. Bosker, J. W. Bosmans, M. C. Boute, N. D. Bouvy, H. Bouwman, A. Brandt-Kerkhof, D. J. Brinkman, S. Bruin, E. R. J. Bruns, J. P. M. Burbach, J. W. A. Burger, C. J. Buskens, S. Clermonts, P. P. L. O. Coene, C. Compaan, E. C. J. Consten, T. Darbyshire, S. M. L. de Mik, E. J. R. de Graaf, I. de Groot, R. J. L. de vos tot Nederveen Cappel, J. H. W. de Wilt, J. van der Wolde, F. C. den Boer, J. W. T. Dekker, A. Demirkiran, M. Derkx-Hendriksen, F. R. Dijkstra, P. van Duijvendijk, M. S. Dunker, Q. E. Eijsbouts, H. Fabry, F. Ferenschild, J. W. Foppen, M. F. Gerhards, P. Gerven, J. A. H. Gooszen, J. A. Govaert, W. M. U. Van Grevenstein, R. Haen, J. J. Harlaar, E. Harst, K. Havenga, J. Heemskerk, J. F. Heeren, B. Heijnen, P. Heres, C. Hoff, W. Hogendoorn, P. Hoogland, A. Huijbers, P. Janssen, A. C. Jongen, F. H. Jonker, E. G. Karthaus, A. Keijzer, J. M. A. Ketel, J. Klaase, F. W. H. Kloppenberg, M. E. Kool, R. Kortekaas, P. M. Kruyt, J. T. Kuiper, B. Lamme, J. F. Lange, T. Lettinga, D. J. Lips, F. Logeman, M. F. Lutke Holzik, E. Madsen, A. Mamound, C. C. Marres, I. Masselink, M. Meerdink, A. G. Menon, J. S. Mieog, D. Mierlo, G. D. Musters, G. A. P. Nieuwenhuijzen, P. A. Neijenhuis, J. Nonner, M. Oostdijk, P. M. P. Paul, K. C. M. J. Peeters, I. T. A. Pereboom, F. Polat, P. Poortman, M. Raber, B. M. M. Reiber, R. J. Renger, C. C. van Rossem, H. J. Rutten, A. Rutten, R. Schaapman, M. Scheer, L. Schoonderwoerd, N. Schouten, A. M. Schreuder, W. H. Schreurs, G. A. Simkens, G. D. Slooter, H. C. E. Sluijmer, N. Smakman, R. Smeenk, H. S. Snijders, D. J. A. Sonneveld, B. Spaansen, E. J. Spillenaar Bilgen, E. Steller, W. H. Steup, C. Steur, E. Stortelder, J. Straatman, H. A. Swank, C. Sietses, H. A. ten Berge, H. G. ten hoeve, W. W. ter Riele, I. M. Thorensen, B. Tip-Pluijm, B. R. Toorenvliet, L. Tseng, J. B. Tuynman, J. van Bastelaar, S. C. van Beek, A. W. H. van de Ven, M. A. J. van de Weijer, C. van den Berg, I. van den Bosch, J. D. W. van der Bilt, S. J. van der Hagen, R. van der hul, G. van der Schelling, A. van der Spek, N. van der Wielen, E. van duyn, C. van Eekelen, J. A. van Essen, K. van Gangelt, A. A. W. van Geloven, C. van Kessel, Y. T. van Loon, A. van Rijswijk, S. J. van Rooijen, T. van Sprundel, L. van Steensel, W. F. van Tets, H. L. van Westreenen, S. Veltkamp, T. Verhaak, P. M. Verheijen, L. Versluis-Ossenwaarde, S. Vijfhuize, W. J. Vles, S. C. Voeten, F. J. Vogelaar, W. W. Vrijland, E. Westerduin, M. E. Westerterp, M. Wetzel, K. P. Wevers, B. Wiering, C. D. M. Witjes, M. W. Wouters, S. T. K. Yauw, E. S. van der Zaag, E. C. Zeestraten, D. D. Zimmerman, T. Zwieten

**Affiliations:** 1 Department of Surgery, Amsterdam UMC, University of Amsterdam, Amsterdam, The Netherlands; 2 Scientific Bureau of the Dutch Institute of Clinical Auditing, Leiden, The Netherlands; 3 Department of Surgery, University Medical Center Groningen, Groningen, The Netherlands; 4 Department of Radiotherapy, Leiden University Medical Center, Leiden, The Netherlands; 5 Department of Radiology, Netherlands Cancer Institute-Antoni van Leeuwenhoek, Amsterdam, The Netherlands; 6 Department of Surgery, Isala Hospital Zwolle, Zwolle, The Netherlands

## Abstract

**Background:**

The time interval between CRT and surgery in rectal cancer patients is still the subject of debate. The aim of this study was to first evaluate the nationwide use of restaging magnetic resonance imaging (MRI) and its impact on timing of surgery, and, second, to evaluate the impact of timing of surgery after chemoradiotherapy (CRT) on short- and long-term outcomes.

**Methods:**

Patients were selected from a collaborative rectal cancer research project including 71 Dutch centres, and were subdivided into two groups according to time interval from the start of preoperative CRT to surgery (< 14 and ≥ 14 weeks).

**Results:**

From 2095 registered patients, 475 patients received preoperative CRT. MRI restaging was performed in 79.4% of patients, with a median CRT–MRI interval of 10 weeks (interquartile range [IQR] 8–11) and a median MRI–surgery interval of 4 weeks (IQR 2–5). The CRT–surgery interval groups consisted of 224 (< 14 weeks) and 251 patients (≥ 14 weeks), and the long-interval group included a higher proportion of cT4 stage and multivisceral resection patients. Pathological complete response rate (*n* = 34 [15.2%] vs. *n* = 47 [18.7%], *p* = 0.305) and CRM involvement (9.7% vs. 15.9%, *p* = 0.145) did not significantly differ. Thirty-day surgical complications were similar (20.1% vs. 23.1%, *p* = 0.943), however no significant differences were found for local and distant recurrence rates, disease-free survival, and overall survival.

**Conclusions:**

These real-life data, reflecting routine daily practice in The Netherlands, showed substantial variability in the use and timing of restaging MRI after preoperative CRT for rectal cancer, as well as time interval to surgery. Surgery before or after 14 weeks from the start of CRT resulted in similar short- and long-term outcomes.

**Electronic supplementary material:**

The online version of this article (10.1245/s10434-018-07097-7) contains supplementary material, which is available to authorized users.

In the treatment of patients with locally advanced rectal cancer, preoperative chemoradiotherapy (CRT) has proven to reduce the local recurrence (LR) rate significantly and has become the standard of care.^1^ A pathological complete response (pCR) following CRT was found in 15–20% of patients, and this is associated with a favorable oncological outcome.^2^ Furthermore, there is a recent trend towards rectal-preserving treatment in patients with a good response.^3^ One of the unresolved issues regarding preoperative CRT is the optimal time interval to total mesorectal excision (TME) surgery and the role of re-staging in determining this interval. A recent systematic review including 13 studies with a total of 19,652 patients concluded that an interval of ≥ 8 weeks from the end of CRT is safe and efficacious because of higher pCR rates, without increasing complication rates;^4^ however, reasons for delaying surgery in the mostly non-randomized comparisons were not provided, and data on the use and outcome of restaging were lacking. Furthermore, no impact on survival was observed. The GRECCAR-6 trial recently reported contradictory findings.^5^ In this trial, 265 patients from 24 French centers were randomized to between 7 and 11 weeks waiting after the end of CRT. No difference in the pCR rate was observed, but the authors did find a higher morbidity rate and worse quality of the mesorectal resection after a prolonged interval. Restaging was not systematically performed in this trial. It is likely the time interval to surgery has to be tailored to the individual patient, rather than a ‘one size fits all’ approach. Non-responders should continue with further treatment (e.g. surgery or further chemotherapy), and those having a good response might benefit from additional waiting time or consolidation chemotherapy to maximize tumor shrinkage with the highest change of negative resection margins.^6^ However, the role of MRI and 18F-fluorodeoxyglucose-positron emission tomography/computed tomography (FDG-PET/CT) in assessing tumor downstaging after CRT, and its value for clinical decision making, is still unclear.^7^,^8^

The aim of this multicenter, cross-sectional study of patients who underwent CRT for rectal cancer in 71 Dutch centers in 2011 was to first evaluate variation in practice with respect to MRI restaging and its impact on time interval to surgery, and, second, to evaluate the impact of timing of surgery on pathological, surgical, and long-term oncological outcomes.

## Patients and methods

A retrospective, resident-led, collaborative research project with a cross-sectional study design was conducted in 71 of 94 hospitals in The Netherlands by the Dutch Snapshot Research Group (DSRG) in 2015. All patients who underwent resection for primary rectal cancer in these hospitals in 2011 were identified from the Dutch ColoRectal Audit (DCRA).^9^ Additional diagnostic and treatment characteristics, as well as long-term surgical and oncological outcomes, were retrospectively added to the DCRA dataset for the year 2011. Details of this cross-sectional study cohort have been published previously.^10^

### Patients and Definitions

All patients who underwent rectal cancer resection after preoperative CRT between 1 January and 31 December 2011 were selected from the initial cohort. The CRT schedule was not registered in the database but consisted of either 25 fractions of 2 Gy or 28 fractions of 1.8 Gy, with concomitant capecitabine as a radiosensitizer. Only the start date of radiotherapy was available in the dataset. Patients were divided into two interval groups based on the observed median time interval: surgery < 14 weeks from the start of CRT (short interval), and surgery after 14 weeks or more (long interval). Patients with metastasis, patients who received additional neoadjuvant chemotherapy preceding or following CRT, and patients with an unknown start date for CRT were excluded. To evaluate the timing of MRI restaging, the interval between the start of CRT and the date of MRI was categorized into four groups: < 6, 6–8, 8–10, and > 10 weeks (electronic supplementary Fig. 1). The MRI restaging result was classified by the study collaborators as ‘progressive disease’, ‘stable disease’, ‘partial response’ and ‘complete response’ based on the radiology reports. To evaluate the subsequent time interval between MRI restaging and surgery, three groups of < 2, 2–4, and > 4 weeks were defined.

### Outcome Parameters

Pathological outcome parameters included pCR (ypT0N0), near pCR (ypT1N0), and circumferential resection margin (CRM) involvement (tumor-free resection margin ≤ 1 mm). Surgical outcome parameters were 30-day overall and surgical complication rate, postoperative blood transfusion, perineal wound problems after abdominoperineal resection (APR), anastomotic leakage after (low) anterior resection (LAR), chronic presacral sinus, length of stay, and re-intervention- and re-admission rate within 30-days. For long-term oncological outcome, 3-year LR, distant recurrence (DR), disease-free survival (DFS), and overall survival (OS) rates were analyzed.

### Statistical Analysis

Categorical or dichotomous outcomes were expressed as absolute numbers with percentages. Statistical analysis of categorical outcomes between groups was performed using the Pearson Chi square test or Fisher’s exact test, where appropriate. Continuous outcomes were expressed as median with interquartile range (IQR). For intergroup variation, the Mann–Whitney *U* test was used following their distribution, otherwise Student’s *t* test was used for independent samples. To determine the 3-year LR, DR, DFS, and OS rates, the Kaplan–Meier method was used and the log-rank test was used for comparison between the two interval groups. Univariable and multivariable analyses were performed to identify independent predictors for CRM, LR, DR, DFS, and OS. Variables with a *p* value < 0.10 in the univariable analysis were included in the multivariable model. Results of the multivariable analyses were reported as odds ratios (OR) with 95% confidence intervals (CIs). A *p* value < 0.05 was considered statistically significant. Statistical analyses were performed in PASW Statistics version 24 (IBM Corporation, Armonk, NY, USA).

## Results

### Patient Characteristics

Within the total cohort of 2095 patients, 684 patients underwent preoperative CRT. After exclusion of patients with metastasis (*n* = 92), patients who received additional preoperative chemotherapy (*n* = 3) and patients with unknown start date of CRT (*n* = 114), 475 patients remained for inclusion in the present analysis.

**Fig. 1:**
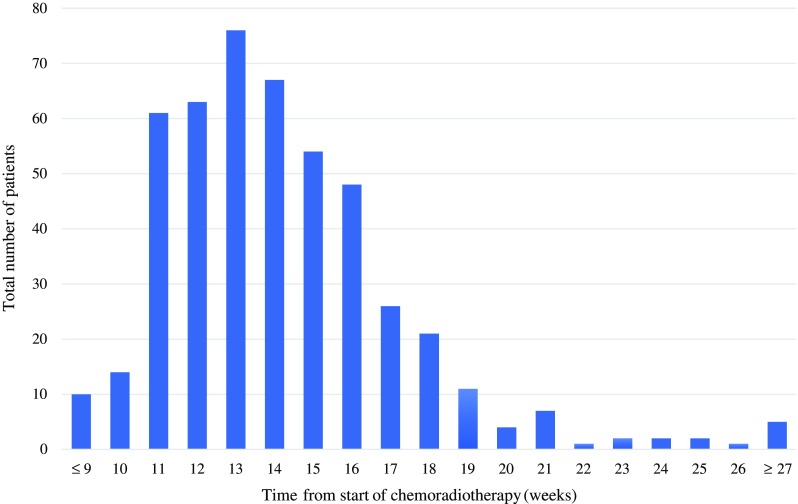
Number of patients for each chemoradiotherapy-surgery time interval in weeks from start of chemoradiotherapy

The median time interval between the start of CRT and surgery was 14 weeks (IQR 12–16) (Fig. 1). Based on this median time interval, patients were subdivided into two groups: 224 patients with a short interval and 251 patients with a long interval. Patient and tumor characteristics of the two groups are reported in Table 1. Significant differences between the interval groups were found for American Society of Anesthesiologists (ASA) score, distance to the anorectal junction on MRI, and clinical tumor stage. Regarding treatment characteristics, the proportion of open approaches (45.5% vs. 58.6%, *p* = 0.011) and multivisceral resections (8.7% vs. 16.5%, *p* = 0.012) was significantly different between the short- and long-interval groups. The proportion of patients receiving adjuvant chemotherapy in the short-interval group was lower in comparison with the long-interval group, however was not statistically significant (*n* = 16 [7.1%] vs. *n* = 31 [12.4%], *p* = 0.058)

**Table 1:** Patient, tumor, and treatment characteristics, and pathological, surgical, and long-term oncologic outcomes for the short- and long-interval groups

	Overall [*n* = 475] (%)	<14 weeks interval [*n* = 224] (%)	≥ 14 weeks interval [*n* = 251] (%)	*p* value
*Sex*				
Male	300/475 (63.2)	140/224 (62.5)	160/251 (63.7)	0.779
*Age, years*				0.169
<60	159/475 (33.5)	84/224 (37.5)	75/251 (29.9)	
61–70	192/475 (40.4)	91/224 (40.6)	101/251 (40.2)	
71–80	110/475 (23.2)	43/224 (19.2)	67/251 (26.7)	
> 80	14/475 (2.9)	6/224 (2.7)	8/251 (3.2)	
*ASA score*				**0.026**
I–II	418/475 (88.0)	205/224 (91.5)	213/251 (84.9)	
III–IV	57/475 (12.0)	19/224 (8.5)	38/251 (15.1)	
*Preoperative imaging* ^*a*^				0.061
MRI	446/465 (96.0)	215/220 (97.7)	231/245 (94.3)	
CT	19/465 (4.0)	5/220 (2.3)	14/245 (5.7)	
*Distance to the anorectal junction, cm*				**0.043**
< 3	143/475 (30.1)	72/224 (32.1)	71/251 (28.2)	
3.1–7.0	129/475 (27.2)	57/224 (25.4)	72/251 (28.7)	
> 7	108/475 (22.7)	60/224 (26.8)	48/251 (19.1)	
Unknown	95/475 (20.0)	35/224 (15.6)	60/251 (23.9)	
*MRF*				
Positive	99/475 (20.8)	47/224 (21.0)	52/251 (20.7)	0.943
*Clinical tumor stage*				
cT3N0M0	59/475 (12.4)	29/224 (12.9)	30/251 (12.0)	0.743
cT4N0M0	15/475 (3.2)	7/224 (3.1)	8/251 (3.2)	0.969
cT1-3N1-2M0	269/475 (56.6)	134/224 (59.8)	135/251 (53.8)	0.185
cT4N1-2M0	49/475 (10.3)	16/224 (7.1)	33/251 (13.1)	**0.032**
Unknown	20/475 (4.2)	13/224 (5.8)	7/251 (2.8)	0.102
*Procedure*				
LAR with primary anastomosis	192/475 (40.4)	100/224 (44.6)	92/251 (36.7)	0.289
APR	202/475 (42.5)	89/224 (39.7)	113/251 (45.0)	
Hartmann	76/475 (16.0)	32/224 (14.3)	44/251 (17.5)	
Other	5/475 (1.1)	3/224 (1.3)	2/251 (0.8)	
*Approach*				**0.011**
Open	249/475 (52.4)	102/224 (45.5)	147/251 (58.6)	
Laparoscopic	220/475 (46.3)	120/224 (53.6)	100/251 (39.8)	
Laparoscopic conversion	6/475 (1.3)	2/224 (0.9)	4/301 (1.6)	
*Multivisceral resection (additional)* ^*b*^				
Yes	60/466 (12.9)	19/218 (8.7)	41/248 (16.5)	**0.012**
No	406/466 (87.1)	199/218 (91.3)	207/248 (83.5)	
*Adjuvant chemotherapy*				
Yes	47/475 (9.9)	16/224 (7.1)	31/251 (12.4)	0.058
*MRI restaging after CRT* ^*c*^				**0.002**
Yes	366/461 (79.4)	158/216 (73.1)	208/245 (84.9)	
No	95/461 (20.6)	58/216 (26.9)	37/245 (15.1)	
*MRI restaging results* ^*d*^				0.244
Progression	7/361 (1.9)	1/7 (14.3)	6/7 (85.7)	
Stable	45/361 (12.5)	19/45 (42.2)	26/45 (57.8)	
Partial response	194/361 (53.7)	130/294 (44.2)	164/294 (55.8)	
Complete response	15/361 (4.2)	4/15 (26.7)	11/15 (73.3)	
*Interval CRT–MRI (weeks)* ^*e*^				**< 0.001**
<6	7/341 (2.0)	5/146 (3.4)	2/195 (1.0)	
6–8	21/341 (6.2)	14/146 (9.6)	7/195 (3.6)	
8–10	114/341 (33.4)	73/146 (50.0)	41/195 (21.0)	
10–12	117/341 (34.3)	47/146 (32.2)	70/195 (35.9)	
12–14	50/341 (14.7)	7/146 (4.8)	43/195 (22.1)	
14–16	21/341 (6.2)	–	21/195 (10.8)	
> 16	13/341 (3.8)	–	11/195 (5.6)	
*Interval MRI–surgery (weeks)* ^*f*^				**< 0.001**
< 1	9/340 (2.6)	6/146 (4.1)	3/194 (1.5)	
1–2	28/340 (8.2)	21/146 (14.4)	7/194 (3.6)	
2–3	63/340 (18.5)	43/146 (29.5)	20/194 (10.3)	
3–4	69/340 (20.3)	36/146 (24.7)	33/194 (17.0)	
4–5	62/340 (18.2)	24/146 (16.4)	38/194 (19.6)	
5–6	41/340 (12.0)	13/146 (8.9)	28/194 (14.4)	
6–7	25/340 (7.4)	3/146 (2.1)	22/194 (11.3)	
7–8	17/340 (5.0)	–	17/194 (8.8)	
8–9	8/340 (2.4)	–	8/194 (4.1)	
> 9	18/340 (0.3)	–	18/194 (9.3)	
*Pathological, surgical and long-term oncologic outcomes*				
Histological type tumor^g^				
Adenocarcinoma	431/461 (93.5)	206/219 (94.1)	225/242 (93.0)	0.087
Mucinous	10/461 (2.2)	7/219 (3.2)	3/242 (1.2)	
Signet ring cell	15/461 (0.2)	1/19 (0.5)	14/242 (5.8)	
Other	5/461 (4.1)	5/219 (2.2)	0/242 (0.0)	
CRM^h^				0.145
Positive	47/365(12.9)	17/176 (9.7)	30/189 (15.9)	
Negative	318/365 (87.1)	159/176 (90.3)	159/189 (84.1)	
Unknown	29/365 (8.0)	14/176 (8.0)	15/189 (7.9)	
ypTN classification				
ypT0N0 (pCR)	81/475 (17.0)	34/224 (15.2)	47/251 (18.7)	0.305
ypT1N0 (near pCR)	30/475 (6.3)	17/224 (7.6)	13/251 (5.2)	0.281
ypT0N1-2	9/475 (1.9)	1/224 (0.4)	8/251 (3.2)	**0.029**
Postoperative transfusion^i^				
Yes	59/475 (12.4)	19/222 (8.6)	40/245 (16.3)	**0.021**
Any perineal wound problems				
<1 year (APR)	64/202 (31.7)	33/89 (37.1)	31/113 (27.4)	0.144
Overall leak rate (LAR)^j^	42/192 (21.9)	19/100 (19.0)	23/92 (25.0)	0.329
Chronic sinus rate (LAR)^k^	25/192 (13.0)	11/100 (11.0)	14/92 (15.2)	0.366
30-day overall complication rate^l^	166/471 (35.2)	74/222 (33.3)	92/249 (36.9)	0.445
30-day surgical complication rate	103/475 (21.7)	45/224 (20.1)	58/251 (23.1)	0.943
Length of stay (median [IQR])^m^	8 [6–14]	7 [6–12]	9 [7–15]	0.131
Re-intervention < 30 days	64/475 (13.5)	28/224 (12.5)	36/251 (14.3)	0.557
Re-admission < 30 days	2/475 (0.4)	1/224 (0.5)	1/251 (0.4)	0.211
Follow-up months (median [IQR])^n^	43 [35–47]	43 [36–47]	42 [32–47]	0.349
3-year local recurrence (Kaplan–Meier)	26/475 (5.5)	9/224 (4.0)	17/251 (6.8)	0.169
3-year distant recurrence (Kaplan–Meier)	90/475 (18.9)	45/224 (20.1)	45/251 (17.9	0.769
3-year disease-free survival (Kaplan–Meier)	343/475 (72.2)	167/223 (74.6)	176/251 (70.1)	0.267
3-year overall survival (Kaplan–Meier)^o^	404/474 (85.2)	196/223 (87.9)	208/251 (82.9)	0.178

A *p* value of less than 0.05 was considered statistically significant and it is highlighted in bold

*ASA* American Society of Anesthesiologists, *MRI* magnetic resonance imaging, *CT* computed tomography, *MRF* mesorectal fascia, *LAR* low anterior resection, *APR* abdominoperineal resection, *CRT* chemoradiotherapy, *CRM* circumferential resection margin, *pCR* pathological complete response, *IQR* interquartile range

^a^Preoperative imaging was not reported in 10 patients

^b^Multivisceral resection was not reported in 9 patients

^c^MRI restaging after CRT was not reported in 14 patients

^d^MRI restaging results were not reported in 114 patients

^e^Interval CRT–MRI restaging was not reported in 134 patients

^f^Interval CRT–MRI restaging was not reported in 135 patients

^g^Not reported in 14 patients

^h^CRM involvement is calculated by subtraction of unknown CRM and pCR

^i^Transfusion was not reported in 8 patients

^j^Overall leak rate was not reported in 5 patients

^k^Chronic sinus rate was not reported in 5 patients

^l^30-day overall complication rate was not reported in 4 patients

^m^Length of stay was not reported in 5 patients

^n^Median follow-up was not reported in 3 patients

^o^3-year overall survival was not reported in 1 patient

### Magnetic Resonance Imaging Restaging Results

MRI restaging was performed in 366 patients (79.4%), with a significant difference between the short- and long-interval groups (73.1% and 84.9%, *p* = 0.002). The median interval between the start of CRT and MRI restaging was 10 weeks (IQR 8–11), and the median interval between MRI restaging and surgery was 4 weeks (IQR 2–5). The CRT–MRI and MRI–surgery intervals are displayed in Table 1 for each of the interval groups, and the MRI restaging results are displayed in Table 2 for the different CRT–MRI and MRI–surgery intervals. With regard to MRI restaging results and pCR rate, a significant association was found for patients with a complete response in comparison with the other MRI restaging results (*n* = 7 [46.7%], *p* = 0.022). No significant association between the restaging result and the timing of MRI restaging was found.

**Table 2:** Interval between start of CRT and MRI restaging, and interval between MRI restaging and surgery

	MRI restaging results				
	Progression (%)	Stable (%)	Partial response (%)	Complete response (%)	*p* value
*CRT*–*MRI restaging interval*^*a*^					
< 6 weeks	0	0	2/2 (100)	0	0.447
6–8 weeks	0	5/32 (15.6)	27/32 (84.4)	0	
8–10 weeks	1/121 (0.8)	18/121 (14.9)	99/121 (81.8)	3/121 (2.5)	
> 10 weeks	6/183 (3.3)	18/183 (9.8)	148/183 (80.9)	11/183 (6.0)	
Overall	7/338 (2.1)	41/338 (12.1)	276/338 (81.7)	14/338 (4.1)	
*MRI restaging*—*surgery interval*^*b*^					
Time interval, weeks (median [IQR])	3 [2–4]	4.5 [3–6]	3 [2–5]	3.5 [2–4]	0.598
< 2 weeks	1/7 (14.3)	4/40 (10)	29/276 (10.5)	2/14 (14.2)	
2–4 weeks	3/7 (42.9)	10/40 (25)	113/276 (40.9)	5/14 (35.7)	
> 4 weeks	3/7 (42.9)	26/40 (65)	134/276 (48.6)	7/14 (50.0)	
pCR	1/7 (14.3)	6/40 (15)	49/276 (17.8)	7/14 (50.0)	0.022

*p* values were calculated for the total study group

*CRT* chemoradiotherapy, *MRI* magnetic resonance imaging, *IQR* interquartile range, *pCR* pathological complete response

^a^CRT–MRI restaging interval results were not reported in 137 patients

^b^MRI restaging-surgery interval results were not reported in 138 patients

### Pathological and Surgical Outcomes

No statistically significant differences were found in the pCR rate (*n* = 34 [15.2%] vs. *n* = 47 [18.7%], *p* = 0.305) and near pCR rate (*n* = 17 [7.6%] vs. *n* = 13 [5.2%], *p* = 0.373) between the short- and long-interval groups (Table 1). Significantly less-isolated residual nodal disease (ypT0N1-2) was found in the short-interval group (*n* = 1 [0.4%] vs. *n* = 8 [3.2%], *p* = 0.029). The proportion of CRM involvement was lower after a short interval, but did not reach statistical significance (*n* = 17 [9.7%] vs. *n* = 30 [15.9%], *p* = 0.145). With regard to postoperative outcomes, significantly less patients received postoperative transfusion in the short-interval group (*n* = 19 [8.6%] vs. *n* = 40 [16.3%], *p* = 0.021). No significant differences were found between the two interval groups in regard to length of stay (median 7 vs. 9 days, *p* = 0.131), perineal wound problems < 1 year following APR (*n* = 33 [37.1%] vs. *n* = 31 [27.4%], *p* = 0.144), overall anastomotic leakage rate following LAR (*n* = 19 [19.0%] vs. *n* = 23 [25.0%], *p* = 0.329), and chronic sinus rate (*n* = 11 [11.0%], *n* = 14 [15.2%], *p* = 0.366).

### Long-Term Oncologic Outcomes

**Fig. 2:**
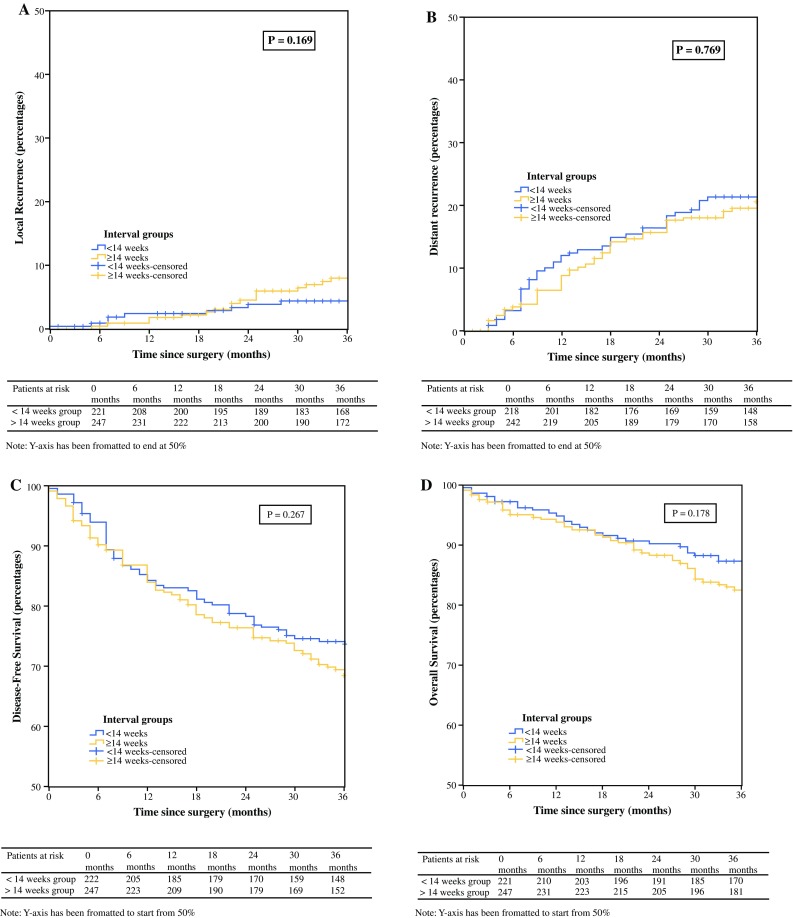
Kaplan–Meier of local recurrence, distant recurrence, disease-free survival and overall survival in short- and long interval group

The median long-term follow-up was 43 months (IQR 35–47) and was similar between the two interval groups (Table 1). With regard to LR rates (*n* = 9 [4.0%] vs. *n* = 17 [6.8%], *p* = 0.169) (Fig. 2a) and DR rates (*n* = 45 [20.1%] vs. *n* = 45 [17.9%], *p* = 0.769) (Fig. 2b), no significant differences were found between the short- and long-interval groups, respectively. Similarly, no significant differences were found for DFS (*n* = 167 [74.6%] vs. 176 [70.1%] *p* = 0.267) (Fig. 2c) and OS (*n* = 196 [87.9%] vs. *n* = 208 [82.9%], *p* = 0.178) (Fig. 2d).

### Predictors of Circumferential Resection Margin Involvement, Recurrence, and Survival

The results of univariable and multivariable analyses for CRM involvement are shown in Table 3. In multivariable analysis, laparoscopic conversion, intraoperative complications, and nodal stage were identified as independent predictors for CRM involvement. Five of the six patients in whom a laparoscopic resection was converted to an open approach had a positive CRM (one patient and four patients for the short- and long-interval groups, respectively). The results of the univariable and multivariable analysis for LR, DR, DFS, and OS are provided in electronic supplementary Tables 1 and 2. The time interval between the start of CRT and surgery was not associated with any of these outcome parameters.

**Table 3:** Univariable and multivariable analyses for CRM

Variable	Univariable analysis		Multivariable analysis	
	OR (95% CI)	*p* value	OR (95% CI)	*p* value
*Sex*				
Male	1.15 (0.61–2.16)	0.675		
Female	Ref			
*BMI, kg/m* ^*2*^				
< 25	0.72 (0.37–1.38)	0.319		
25–30	Ref			
> 30	0.85 (0.35–2.08)	0.724		
*Distance to the anorectal junction, cm*				
< 3	0.82 (0.33–2.00)	0.658		
3.1–7	1.10 (0.46–2.61)	0.832		
> 7	Ref			
*Approach*				
Open	Ref		Ref	
Laparoscopic	**0.54 (0.28–1.05)**	**0.068**	0.56 (0.27–1.15)	0.113
Laparoscopic conversion	**39.46 (4.45–350)**	**0.001**	**39.1 (3.87–395)**	**0.002**
*Intraoperative complication*				
Yes	**3.65 (1.11–11.97)**	**0.033**	**5.22 (1.43–19.11)**	**0.013**
*Multiple visceral resection*				
Yes	**2.31 (1.10–4.83)**	**0.026**	1.98 (0.75–5.26)	0.170
*Tumor stage*				
ypT0	Ref		Ref	
ypT1-3	**3.40 (1.02–11.28)**	**0.046**	2.62 (0.74–9.27)	0.135
ypT4	**8.46 (2.03–35.20)**	**0.003**	2.14 (0.39–11.79)	0.381
*Nodal stage*				
ypN0	Ref		Ref	
ypN1-2	**3.47 (1.87–6.41)**	**< 0.001**	**3.13 (1.60–6.16)**	**0.001**
*Interval group*				
Short	Ref			
Long	1.65 (0.89–3.09)	0.115		

Variables with a *p* value less than 0.10 in the univariable analysis were included in the multivariable model. A *p* value of less than 0.05 was considered statistically significant and it is highlighted in bold

*OR* odds ratio, *CI* confidence interval, *CRM* circumferential resection margin, *BMI* body mass index

## Discussion

This large cross-sectional study including 71 Dutch centers reflects daily practice in The Netherlands in 2011 with regard to clinical management of locally advanced rectal cancer. MRI restaging after CRT was performed in 79% of patients, with substantial variability in timing. In addition, substantial variability in the time interval between the start of CRT and surgery was observed, with a median of 14 weeks. Using the median interval as the cut-off, similar postoperative and long-term surgical and oncological outcomes were found for the two interval groups. The median of 14 weeks after the start of CRT corresponds with approximately 9 weeks after the end of CRT, which is between the two intervals to which patients were randomized in the GRECCAR-6 trial (7 vs. 11 weeks). Despite the methodological shortcomings in this comparison, we could not confirm the unfavorable results after longer waiting times in the GRECCAR-6 trial.

The Dutch rectal cancer guideline from 2008, still being used in 2011, did not include a statement on the use and interpretation of restaging MRI after CRT for rectal cancer, nor did it recommend a certain time interval for response evaluation or surgery following CRT. This likely explains the large observed variability in practice. A significantly lower proportion of MRI restaging in the short-interval group (73% vs. 85%) was likely related to some hospitals that routinely planned surgery after 6–8 weeks from the end of radiotherapy without any response assessment, thereby following the guideline at that time.

Standardized MRI assessment regarding tumor regression grade (mrTRG) has been proposed, and good interobserver agreement can be achieved after interactive case-based learning.^11^ Although mrTRG correlates with oncological outcome, the role of MRI in restaging rectal cancer after CRT as a single diagnostic modality, and its clinical implications, are still the subject of debate.^12^ When restaging MRI suggested complete tumor response in the present study, pathology revealed cancer in 50% of patients in the present study, confirming the reported inaccuracy of restaging MRI.^13^ Nowadays, the impact of restaging MRI is likely to be different. The European Society of Gastrointestinal and Abdominal Radiology (ESGAR) recently published their updated recommendations.^14^ Routinely adding diffusion-weighted MRI for the purpose of yT restaging reached consensus, especially due to improved differentiation between partial and complete response. Structured MRI reporting was unanimously recommended and a template for both primary and restaging was published. Following such consensus guidelines will likely reduce hospital variation and increase the clinical impact of MRI restaging.

Nowadays, response evaluation and timing of subsequent treatment after CRT is often aimed at identifying clinical complete responders who are candidates for a watch-and-wait policy. Digital examination and endoscopy are also important modalities, besides MRI restaging, in this clinical scenario. However, patients included in the present study were treated at the time a watch-and-wait strategy was considered experimental, with only a single institution in The Netherlands publishing their initial experience.^15^ Other implications of restaging MRI might be limiting the extent of the resection in order to refrain from multivisceral resection or enable sphincter preservation. Unfortunately, the dataset did not include variables to analyze these outcomes.

The optimal length of the interval between neoadjuvant CRT and surgery has frequently been analyzed, but with conflicting results regarding pathological response.^16^–^24^ It has been suggested that tumor response to CRT can take up to several months, depending on tumor volume and characteristics.^25^,^26^ Only a few randomized controlled trials compared short and long intervals between CRT and surgery, with conflicting results in relation to pCR rates.^27^,^28^ Most recently, the GRECCAR-6 trial showed no differences in pCR rates between 7- and 11-week interval.^5^ Similarly, pCR rates did not differ between the two intervals in the present study (15.2% vs. 18.7%); however, even with a higher clinical stage at baseline, pCR rates were slightly higher in the long-interval group. The significantly higher proportion of patients with cT4N1-2 stage in the long-interval group (13% vs. 7%) was likely the reason for a higher proportion of multivisceral resections (17% vs. 9%). As a consequence, this probably also explains the higher proportion of CRM positivity (16% vs. 10%) and isolated residual nodal disease (ypT0N1-2) in the long-interval group, as well as more postoperative blood transfusions (16% vs. 9%). The GRECCAR-6 trial suggested that longer waiting times after radiotherapy increase the difficulty of surgery due to more radiation-induced fibrosis, probably explaining the observed higher morbidity rates and worse quality of the specimen in that study. This might have also been an explanation for the higher CRM positivity and blood transfusion rates after longer waiting times in the present study.

Previous studies comparing the long-term oncologic outcomes for different time intervals between CRT and surgery have shown conflicting results.^29^–^31^ In the follow-up of the Lyon R90-01 trial, the study group found no significant differences between the two intervals regarding LR rates and OS (5-, 10-, 15-, and 17-year follow-up).^32^ Some studies demonstrated improved prognosis after longer intervals.^33^,^34^ We could not find any impact on the oncological outcome of waiting times between CRT and surgery, which is consistent with other retrospective studies.^35^ Pathological tumor and nodal status, CRM involvement, and multivisceral resection were identified as significant predictors of long-term oncological outcomes in this cohort of locally advanced rectal cancer patients with neoadjuvant CRT, whereas adjuvant chemotherapy was not associated with DFS and OS. The Dutch rectal cancer guideline from 2008 did not recommend adjuvant chemotherapy for stage 3 rectal cancer, and this has not been changed in the guideline revision of 2014 because this is still not considered to prolong survival.^36^,^37^ This explains the low number of patients in both study groups who received adjuvant chemotherapy.

The strength of this cross-sectional study is the large population-based data with short- and long-term intervals to surgery, reflecting daily practice. However, limitations of this study design are related to the retrospective data collection, with, for example, missing information on patient-tailored approaches or institutional protocols. Excluding patients without a reported start date for CRT could have resulted in selection bias. MRI restaging results were pragmatically categorized into four options for retrospective interpretation of the MRI reports, while central review using standardized criteria, as recently published by ESGAR, would have been more informative. Furthermore, not all Dutch hospitals participated in this voluntary cross-sectional study, in contrast to the mandatory DCRA, which might limit the representativeness of the results. Finally, patients who did not proceed to surgery after CRT because of a watch-and-wait policy, treatment-related toxicity, or other reasons are not recorded in this cross-sectional study as only patients who underwent resection were included.

## Conclusions

This large cross-sectional study on CRT followed by TME surgery for locally advanced rectal cancer, reflecting daily practice in The Netherlands in 2011, showed high usage of restaging MRI not supported by guideline recommendations at that time. Timing of surgery after preoperative CRT was highly variable, partially related to the variable impact of MRI-based response assessment and differences in patient and tumor characteristics, however this did not significantly influence short- and long-term outcomes.

## Electronic supplementary material

Below is the link to the electronic supplementary material.

Supplementary material 1 (DOCX 1851 kb)

Supplementary material 2 (DOCX 49 kb)

## Appendix

### Definitions

Low anterior resection with primary anastomosis (LAR) was defined as a TME, with or without diverting stoma. APR was defined, according to the TME principles, as a rectal resection including the anal sphincter complex with a definitive colostomy. The low Hartmann’s procedure was defined as an LAR with rectal stump closure and a definitive colostomy.

LR was defined as the recurrent disease at the anastomotic site, in the pelvis, or in the perineal wound. DR was defined as metastatic localizations, not present at primary rectal cancer surgery. DFS was defined as all patients without recurrent disease, excluding the patients lost to follow-up. OS was defined as all patients alive after the end of follow-up, excluding the patients lost to follow-up.
