# Supplementary material for: Cross-Sectional Study on MRI Restaging After Chemoradiotherapy and Interval to Surgery in Rectal Cancer: Influence on Short- and Long-Term Outcomes
Source: Ann Surg Oncol. 2018 Dec 13;26(2):437–48. doi: 10.1245/s10434-018-07097-7 (PMC6341052; doi:10.1245/s10434-018-07097-7)
Supplement: Supplementary file 1 — Supplementary material 1 (DOCX 1851 kb) [file 10434_2018_7097_MOESM1_ESM.docx]

**
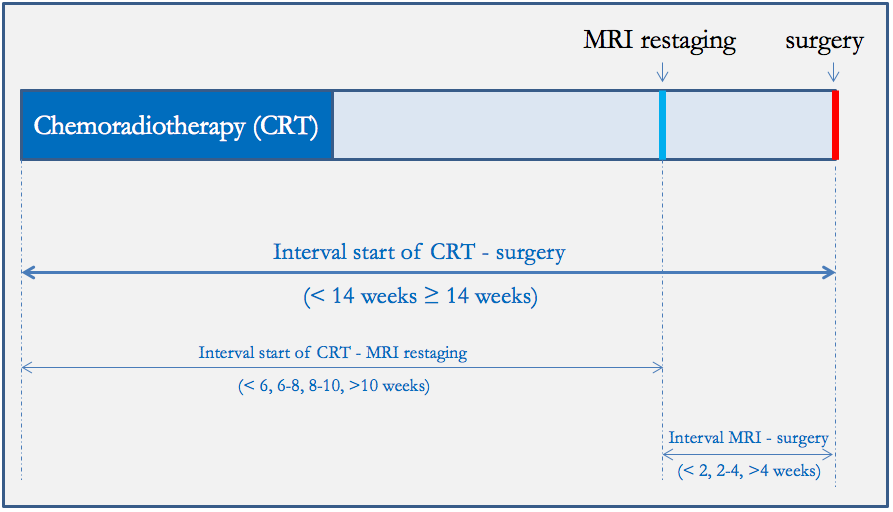
**

**SUPPLEMENT FIG 1.** The intervals used in this study, between start of CRT and surgery, start of CRT and MRI-restaging and MRI-restaging and surgery. *MRI* Magnetic Resonance Imaging, *CRT* Chemoradiotherapy
