# Supplementary material for: Cross-Sectional Study on MRI Restaging After Chemoradiotherapy and Interval to Surgery in Rectal Cancer: Influence on Short- and Long-Term Outcomes
Source: Ann Surg Oncol. 2018 Dec 13;26(2):437–48. doi: 10.1245/s10434-018-07097-7 (PMC6341052; doi:10.1245/s10434-018-07097-7)
Supplement: Supplementary file 2 — Supplementary material 2 (DOCX 49 kb) [file 10434_2018_7097_MOESM2_ESM.docx]

**SUPPLEMENT TABLE 1.** Uni- and multivariable analyses for LR and DR.

|  |  | **Local Recurrence** | | | | **Distant Recurrence** | | | |
| --- | --- | --- | --- | --- | --- | --- | --- | --- | --- |
| Variable |  | Univariable analysis | | Multivariable analysis | | Univariable analysis | Multivariable analysis | | |
|  |  | OR  (95% CI) | P-value | OR  (95% CI) | P-value | OR  (95% CI) | P-value | OR  (95% CI) | P-value |
| Gender | Male | 1.38 (0.60-3.16) | 0.454 |  |  | 1.16 (0.75-1.79) | 0.513 |  |  |
| Age | <60 | Ref |  |  |  | Ref |  |  |  |
|  | 61-70 | **0.41 (0.16-1.10)** | **0.077** |  | NS | **0.63 (0.39-1.03)** | **0.067** |  | NS |
|  | 71-80 | 0.79 (0.30-2.11) | 0.638 |  |  | 0.85 (0.50-1.46) | 0.565 |  |  |
|  | >80 | 2.20 (0.49-9.85) | 0.301 |  |  | 1.02 (0.31-3.30) | 0.978 |  |  |
| BMI | <25 | 0.86 (0.38-1.91) | 0.702 |  |  | 0.84 (0.53-1.31) | 0.434 |  |  |
|  | 25-30 | Ref |  |  |  | Ref |  |  |  |
|  | >30 | 0.44 (0.10-1.95) | 0.281 |  |  | 0.84 (0.44-1.59) | 0.591 |  |  |
| ASA score | I/II | Ref |  |  |  | Ref |  |  |  |
|  | III/IV | 1.20 (0.36-4.009) | 0.766 |  |  | 1.31 (0.70-2.46) | 0.406 |  |  |
| Distance anorectal junction | <3cm | **2.60 (0.85-7.98)** | **0.095** | **3.47 (1.04-11.51)** | **0.042** | 1.54 (0.83-2.87) | 0.176 |  |  |
|  | 3.1-7cm | 0.46 (0.08-2.49) | 0.363 |  |  | 1.41 (0.73-2.72) | 0.302 |  |  |
|  | >7cm | Ref |  |  |  | Ref |  |  |  |
| Tumor stage | ypT0 | Ref |  |  |  | Ref |  |  |  |
|  | ypT1-3 | 2.17 (0.50-9.38) | 0.301 |  |  | **4.15 (1.68-10.25)** | **0.002** | **2.76 (1.10-6.93)** | **0.031** |
|  | ypT4 | **10.68 (2.22-51.44)** | **0.003** |  | NS | **5.16 (1.69-15.76)** | **0.004** |  | NS |
| Nodal stage | ypN0 | Ref |  |  |  | Ref |  |  |  |
|  | ypN1-2 | **3.31 (1.52-7.20)** | **0.003** | **9.01 (2.82-28.81)** | **<0.001** | **3.68 (2.42-5.61)** | **<0.001** | **3.09 (1.97-4.84)** | **<0.001** |
| CRM | Positive | **4.04 (1.70-9.61)** | **0.002** |  | NS | **2.82 (1.64-4.85)** | **<0.001** | **1.80 (1.03-3.15)** | **0.041** |
|  | Negative | Ref |  |  |  | Ref |  |  |  |
| Multivisceral resection | Yes | **2.65 (1.05-6.67)** | **0.039** |  | NS | **2.49 (1.52-4.06)** | **<0.001** | **2.54 (1.47-4.39)** | **0.001** |
| Approach | Open | Ref |  |  |  | Ref |  |  |  |
|  | Lap. | 1.15 (0.51-2.55) | 0.740 |  |  | 0.97 (0.64-1.48) | 0.903 |  |  |
|  | Lap. conversion | **8.22 (1.84-36.78)** | **0.006** |  | NS | 1.13 (0.16-8.19) | 0.904 |  |  |
| Postoperative complication | Yes | 1.00 (0.45-2.25) | 0.996 |  |  | 0.97 (0.62-1.51) | 0.888 |  |  |
| Surgical complication | Yes | 4.45 (0.56-35.55) | 0.160 |  |  | 1.07 (0.50-2.30) | 0.865 |  |  |
| Postoperative transfusion | Yes | 1.48 (0.51-4.30) | 0.475 |  |  | 1.48 (0.84-2.62) | 0.178 |  | NS |
| Interval group | Short | Ref |  |  |  | Ref |  |  |  |
|  | Long | 1.75 (0.78-3.93) | 0.175 |  |  | 0.94 (0.62-1.42) | 0.771 |  |  |

*OR* Odds Ratio, *CI* Confidence Interval, *BMI* Body Mass Index, *ASA* American Society of Anaesthesiologists-Classification, *CRM* Circumferential Resection Margin, *Ref* reference, *NS* not significant.

**SUPPLEMENT TABLE 2.** Uni- and multivariable analyses for DFS and OS.

|  |  | **Disease Free Survival** | | | | | **Overall Survival** | | | |
| --- | --- | --- | --- | --- | --- | --- | --- | --- | --- | --- |
| Variable |  | Univariable analysis | | Multivariable analysis | | | Univariable analysis | | Multivariable analysis | |
|  |  | OR  (95% CI) | P-value | | OR  (95% CI) | P-value | OR  (95% CI) | P-value | OR  (95% CI) | P-value |
| Gender | Male | 1.22 (0.85-1.75) | 0.283 | |  |  | 0.83 (0.44-1.59) | 0.578 |  |  |
| Age | <60 | Ref |  | |  |  | Ref |  |  |  |
|  | 61-70 | 0.81 (0.54-1.21) | 0.308 | |  |  | 0.68 (0.33-1.40) | 0.292 |  |  |
|  | 71-80 | 1.03 (0.65-1.61) | 0.913 | |  |  | 0.80 (0.34-1.87) | 0.609 |  |  |
|  | >80 | 1.32 (0.53-3.32) | 0.555 | |  |  | 0.82 (0.11-6.19) | 0.847 |  |  |
| BMI | <25 | 0.78 (0.54-1.13) | 0.185 | |  |  | **0.50 (0.24-1.03)** | **0.061** |  | NS |
|  | 25-30 | Ref |  | |  |  | Ref |  |  |  |
|  | >30 | 0.82 (0.49-1.39) | 0.462 | |  |  | 0.66 (0.25-1.75) | 0.407 |  |  |
| ASA score | I/II | Ref |  | |  |  | Ref |  |  |  |
|  | III/IV | **1.76 (1.10-2.80)** | **0.018** | |  | NS | 0.82 (0.25-2.66) | 0.737 |  |  |
| Distance anorectal junction | <3cm | **1.79 (1.05-3.08)** | **0.034** | | **2.00 (1.14-3.49)** | **0.015** | **2.55 (0.94-6.97)** | **0.067** | **3.40 (1.17-9.92)** | **0.025** |
|  | 3.1-7cm | **1.72 (0.99-3.01)** | **0.057** | | **1.85 (1.05-3.26)** | **0.035** | 1.66 (0.56-4.96) | 0.363 |  |  |
|  | >7cm | Ref |  | |  |  | Ref |  |  |  |
| Tumor stage | ypT0 | Ref |  | |  |  | Ref |  |  |  |
|  | ypT1-3 | **2.38 (1.31-4.33)** | **0.004** | |  | NS | **8.40 (1.15-61.41)** | **0.036** |  | NS |
|  | ypT4 | **3.45 (1.58-7.57)** | **0.002** | |  | NS | **11.85 (1.32-106)** | **0.027** |  | NS |
| Nodal stage | ypN0 | Ref |  | |  |  | Ref |  |  |  |
|  | ypN1-2 | **2.84 (2.01-3.99)** | **<0.001** | | **2.48 (1.61-3.82)** | **<0.001** | **3.67 (1.92-7.04)** | **<0.001** | **3.26 (1.49-7.15)** | **0.003** |
| CRM | Positive | **3.03 (2.00-4.61)** | **<0.001** | | **2.48 (1.47-4.20)** | **0.001** | **5.08 (2.56-10.08)** | **<0.001** | **4.42 (1.93-10.16)** | **<0.001** |
|  | Negative | Ref |  | |  |  | Ref |  |  |  |
| Multivisceral resection | Yes | **2.27 (1.49-3.45)** | **<0.001** | | **2.41 (1.37-4.24)** | **0.002** | **2.57 (1.21-5.46)** | **0.014** | **3.39 (1.38-8.31)** | **0.008** |
| Adjuvant chemotherapy | Yes | 1.01 (0.58-1.76) | 0.961 | |  |  | 1.28 (0.50-3.28) | 0.607 |  |  |
| Approach | Open | Ref |  | |  |  |  |  |  |  |
|  | Lap. | 1.06 (0.75-1.49) | 0.757 | |  |  | 1.22 (0.64-2.33) | 0.542 |  |  |
|  | Lap. conversion | 2.21 (0.69-7.02) | 0.180 | |  |  | 2.76 (0.37-20.7) | 0.323 |  |  |
| Any postoperative complication | Yes | 1.22 (0.86-1.73) | 0.268 | |  |  | 0.99 (0.51-1.94) | 0.982 |  |  |
| Surgical complication | Surgical | 1.04 (0.58-1.85) | 0.903 | |  |  | 0.47 (0.16-1.39) | 0.172 |  |  |
| Postoperative transfusion | Yes | **1.57 (0.99-2.48)** | **0.055** | |  | NS | 1.18 (0.46-3.02) | 0.730 |  |  |
| Interval group | Short | Ref |  | |  |  | Ref |  |  |  |
|  | Long | 1.21 (0.86-1.71) | 0.270 | |  | NS | 0.60 (0.32-1.16) | 0.127 |  |  |

*OR* Odds Ratio, *CI* Confidence Interval, *BMI* Body Mass Index, *ASA* American Society of Anaesthesiologists-Classification, *CRM* Circumferential Resection Margin, *Ref* reference, *NS* not significant.
